# Minocycline prevents hypoxia-induced seizures

**DOI:** 10.3389/fncir.2023.1006424

**Published:** 2023-03-22

**Authors:** Isato Fukushi, Keiko Ikeda, Kotaro Takeda, Masashi Yoshizawa, Yosuke Kono, Yohei Hasebe, Mieczyslaw Pokorski, Yasumasa Okada

**Affiliations:** ^1^Faculty of Health Sciences, Aomori University of Health and Welfare, Aomori, Japan; ^2^Clinical Research Center, Murayama Medical Center, Musashimurayama, Japan; ^3^Homeostatic Mechanism Research Unit, Institute of Innovative Research, Tokyo Institute of Technology, Yokohama, Japan; ^4^Faculty of Rehabilitation, School of Health Sciences, Fujita Health University, Toyoake, Japan; ^5^Department of Pediatrics, Faculty of Medicine, University of Yamanashi, Chuo, Japan; ^6^Institute of Health Sciences, University of Opole, Opole, Poland

**Keywords:** microglia, hypoxia, seizure, SUDEP, minocycline

## Abstract

Severe hypoxia induces seizures, which reduces ventilation and worsens the ictal state. It is a health threat to patients, particularly those with underlying hypoxic respiratory pathologies, which may be conducive to a sudden unexpected death in epilepsy (SUDEP). Recent studies provide evidence that brain microglia are involved with both respiratory and ictal processes. Here, we investigated the hypothesis that microglia could interact with hypoxia-induced seizures. To this end, we recorded electroencephalogram (EEG) and acute ventilatory responses to hypoxia (5% O_2_ in N_2_) in conscious, spontaneously breathing adult mice. We compared control vehicle pre-treated animals with those pre-treated with minocycline, an inhibitory modulator of microglial activation. First, we histologically confirmed that hypoxia activates microglia and that pre-treatment with minocycline blocks hypoxia-induced microglial activation. Then, we analyzed the effects of minocycline pre-treatment on ventilatory responses to hypoxia by plethysmography. Minocycline alone failed to affect respiratory variables in room air or the initial respiratory augmentation in hypoxia. The comparative results showed that hypoxia caused seizures, which were accompanied by the late phase ventilatory suppression in all but one minocycline pre-treated mouse. Compared to the vehicle pre-treated, the minocycline pre-treated mice showed a delayed occurrence of seizures. Further, minocycline pre-treated mice tended to resist post-ictal respiratory arrest. These results suggest that microglia are conducive to seizure activity in severe hypoxia. Thus, inhibition of microglial activation may help suppress or prevent hypoxia-induced ictal episodes.

## Introduction

A seizure is a paroxysmal alteration of neurologic function caused by excessive hypersynchronous discharge of neurons in the brain, which causes temporary abnormalities in muscle tone or movements, behaviors, sensations, or states of awareness (Stafstrom and Carmant, 2015). Severe hypoxia induces seizures (Miyake et al., 2007; Gillam-Krakauer and Carter, 2012), which reduce ventilation and worsens the ictal state, occasionally resulting in sudden unexpected death in epilepsy (SUDEP) (So, 2008; Sowers et al., 2013; Fukushi et al., 2020). This negative spiral is a health risk to patients with epilepsy and comorbid hypoxic respiratory pathologies, e.g., severe asthmatic attack or sleep apnea (Gullach et al., 2015; Harnod et al., 2017). However, pathophysiological mechanisms of hypoxia-induced seizures and post-ictal ventilatory depression remain unclear. Recent progress in glial physiology suggests that microglia are involved with brain neural processes (Wake et al., 2009; Baalman et al., 2015), neurotransmission (Hoshiko et al., 2012), and synaptic plasticity (Rogers et al., 2011). Microglia are also relevant to both epileptic and respiratory functions (Eyo et al., 2017; Camacho-Hernández et al., 2022). Here, we hypothesize that microglia are involved with the occurrence of hypoxia-induced seizures and hypoxic ventilatory responsiveness. We tested the hypothesis by comparing electroencephalogram (EEG) and ventilatory responses to acute severe hypoxia using minocycline, an inhibitory modulator of microglial activation (Tikka et al., 2001; Garrido-Mesa et al., 2013).

## Materials and methods

### Animal welfare

Experiments were approved by the Animal Experiment Ethics Committee of Murayama Medical Center and complied with guidelines for the Care and Use of Laboratory Animals of the National Research Council of the National Academies (8th edition, revised 2011) and Guiding Principles for the Care and Use of Animals of the Physiological Society of Japan.

### Immunohistochemistry

We first performed immunohistochemical investigation to evaluate whether hypoxia activates microglia and minocycline blocks hypoxia-induced microglial activation in the piriform cortex, which is the crucial epileptogenic site in humans and rodents (Piredda and Gale, 1985; Vismer et al., 2015; Chee et al., 2022). We used eight adult male C57BL/6 mice aged 7.0 ± 0.0 weeks (mean ± SD). There were four groups of mice: with and without minocycline pre-treatment, without and with hypoxic loading (*n* = 2 each). Minocycline (Fuji Pharma, Tokyo, Japan) was diluted in saline, neutralized with sodium hydroxide solution to pH 7.4 (final concentration 11.8 mg/ml), and administered for three consecutive days before the experiment. Mice received 50 mg/kg minocycline twice daily for the first 2 days, and once for the next day (Zheng et al., 2015). Mice without minocycline group received saline as a vehicle in like manner. All injections were intraperitoneal. Mice without hypoxia loading were acclimated to a whole-body plethysmography chamber in room air for 60 min and then left in room air for 30 min. Mice subjected to hypoxia loading were also acclimated to the chamber environment for 60 min in room air and exposed to 7% O_2_ hypoxia (N_2_ balanced) for 30 min, followed by 30 min survival in room air. Then, under deep isoflurane anesthesia, mice were transcardially perfused with 4% paraformaldehyde (PFA)/phosphate buffered solution (PBS), pH 7.4, and brains were extracted. Immunohistochemistry was performed as described previously (Ikeda et al., 2007). To evaluate the cellular morphology of microglia, we immunostained ionized calcium-binding adaptor protein-1 (Iba-1), a constitutively expressed protein in almost all microglia, which is widely used as a microglia marker (Ito et al., 1998). The staining was performed using 1:2,000 anti-Iba-1 antibody (Wako, Neuss, Germany) as the first antibody, 1:1,000 fluorescent conjugated anti-rabbit antibody (Thermo Fisher Scientific, MA, USA) as the second antibody, and 1:2,000 alkaline phosphatase (AP)-conjugated anti-fluorescent antibody as the third antibody. The shape of stained resting microglia is ramified, and that of activated microglia is de-ramified round or ovoidal (Kettenmann et al., 2011; Fernández-Arjona et al., 2019). Signals were detected with nitro blue tetrazolium (NBT)/5-bromo-4-chloro-3-indolyl-phosphate (BCIP; Roche Diagnostics, Basel, Switzerland) as a chromogen. The piriform cortex was photographed with a high-resolution digital camera (DP70, Olympus, Tokyo, Japan) and the morphology of Iba-1-immunoreactive cells was examined.

### Functional evaluation

Twenty adult male C57BL/6 mice aged 7.0 ± 0.0 weeks were used. The mice were divided into vehicle and minocycline groups of 10 each and were pre-treated with saline and minocycline, respectively, in the way as described above in the immunohistochemistry section.

#### EEG recordings

EEG electrodes were implanted as described previously (Fukushi et al., 2016, 2020, 2021; Terada et al., 2016). Briefly, the skull surface was surgically exposed under anesthesia with inhaled isoflurane (3.0%) and intraperitoneally injected pentobarbital (45–50 mg/kg). Then, two miniature screws (diameter 1.2 mm, length 2.0 mm) were inserted as recording electrodes into the skull over the frontal lobes, 2.5 mm posterior to the bregma, the third screw (diameter 1.2 mm, length 2.0 mm) along the midline, 4.5 mm anterior to the bregma, as a ground electrode. Dental resin and adhesive were used to fix the implanted electrodes, along with an additional screw for mounting the head. Screws for EEG electrodes were connected with a single amplifier by vinyl-coated flexible copper wires (O.D. 1.1 mm). The mice appeared to fully recover after 7 days, but the recovery period was extended by additional 4 days. The recording was conducted in the conscious, spontaneously breathing condition. EEG signals were amplified (JB-101J and AB-651J, Nihon Kohden, Tokyo, Japan) and bandpass filtered at 0.08–10,000 Hz. The EEG time signal was transferred to the frequency domain *via* a fast Fourier transform to calculate the power of theta (4–8 Hz) and gamma (35–45 Hz) bands. Each theta and gamma power was averaged across time in room air and during initial augmentation of ventilation in hypoxia before seizure appearance.

#### Ventilation recordings

A whole-body plethysmograph (PLY 310, EMMS, Bordon, UK), consisting of a recording and reference chambers placed inside a transparent acrylic box (size 20 × 20 × 20 cm), was used to non-invasively measure the respiratory flow, as described previously (Pokorski et al., 2014; Fukushi et al., 2016, 2020, 2021). Briefly, the mouse was placed in a pre-calibrated recording chamber (volume 530 ml), which was maintained at 25°C throughout the experiment. The air in the chamber was suctioned at a rate of 250 ml/min using a constant flow generator (MV-6005VP, E.M.P-Japan, Tokyo, Japan). The pressure difference between the recording and reference chambers was measured using a differential pressure transducer (TPF100, EMMS), which was amplified with an amplifier (AIU060, Information and Display Systems, Bordon, UK) and bandpassed at 0.1–20 Hz. Since changes in the respiratory flow are proportional to those in the chamber pressure, the flow was calculated based on the pressure difference between the recording and reference chambers. Tidal volume was calculated for each breath (V_T_; μl/g b.w.) by integrating the respiratory flow, and the counted respiratory rate (RR) was assessed as the number of breaths per min. Minute ventilation (⩒_E_; ml/g/min) was expressed as V_T_ × RR. Respiratory variables were calculated in 1 min epochs. Controlled mixing of N_2_ and air in the acrylic box was done to adjust the chamber O_2_ concentration, which was monitored with an oxygen paramagnetic sensor (OxyStar-100, CWE, PA). An A/D converter (PowerLab4/26, ADInstruments, Colorado Springs, CO) was used to digitize pressure, EEG signals, and O_2_ concentration data simultaneously at a sampling rate of 4,000 Hz. Ventilatory and EEG data were stored on a PC hard disk with LabChart7 software (ADInstruments). Signal processing was performed using MATLAB 2020a (MathWorks, Natick, MA).

The mouse was allowed to acclimate to the experimental setup in room air in the recording chamber for 60 min before the EEG and ventilation measurements began. After the normoxic recordings for 5 min, the gas was switched to 5% O_2_ (N_2_ balanced) for the continuous recording of ventilation for 20 min, followed by a switch back to room air. When respiratory arrest occurred during hypoxia, the gas in the chamber was immediately switched back to room air, and the recording was terminated. The moments of seizure appearance and cessation were determined by careful macroscopic observation of animals and counter-confirmed by EEG activity. The occurrence of respiratory arrest was defined as a point of flattening of the respiratory flow signal. Periods of sniffing, grooming, or licking that deviated from the mouse’s normal breathing pattern were discarded. The periods of seizures, distorting the respiratory flow signals, were discarded as well. The variables recorded in the control group with saline were compared to those in the minocycline intervention group.

### Statistical elaboration

The generalized Wilcoxon test was used to assess differences in the incidence of respiratory arrest between the two groups. The Mann-Whitney U test was used for differences in the time of seizure appearance during hypoxia between the vehicle and minocycline pre-treated groups. The mean values of ⩒_E_, V_T_, RR, theta power, and gamma power were respectively submitted to a two-factor within-subject ANOVA; with two pharmacological conditions (vehicle and minocycline), and two O_2_ conditions (baseline room air and 5% O_2_). When significant interactions were obtained, the *post-hoc* analysis was conducted using Welch’s *t*-test between pharmacological conditions and paired *t*-test to determine the significant differences between the oxygen conditions. The criterion for significance was set at *p* < 0.05. All statistical tests were performed using SPSS 29.0 (IBM, Armonk, NY).

## Results

**Figure 1:**
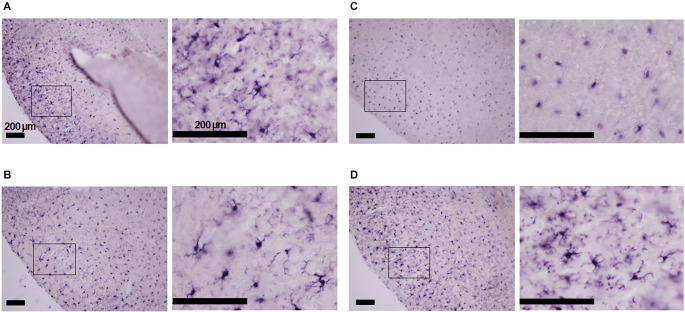
Morphology of Iba-1 positive cells in the piriform cortex of mice in four conditions: with and without minocycline, without and with hypoxic (7% O_2_) loading. **(A)** Room air without minocycline: ramified non-active cells. **(B)** Room air with minocycline: ramified cells. **(C)** Hypoxia without minocycline: activated ovoid cells. **(D)** Hypoxia with minocycline: mostly ramified Iba-1 positive cells intermingled with some ovoid cells. Minocycline suppressed hypoxia-induced microglial activation to some extent in the epileptogenic piriform cortex. The panels’ left-sided rectangles were blown-up in the corresponding right panels. The scale bars are all 200 μm.

The immunohistological investigation showed that the cortical Iba-1 positive cells were ramified in shape in mice breathing room air irrespective of minocycline pre-treatment or its lack (Figures 1A,B). In the hypoxic conditions, Iba-1 positive cells in the vehicle pre-treated mice were ovoidal (Figure 1C). However, those in the minocycline pre-treated mice were mostly ramified but intermingled with a small number of ovoidal cells (Figure 1D). These findings were consistent in the two sets of histological specimens investigated.

**Figure 2:**
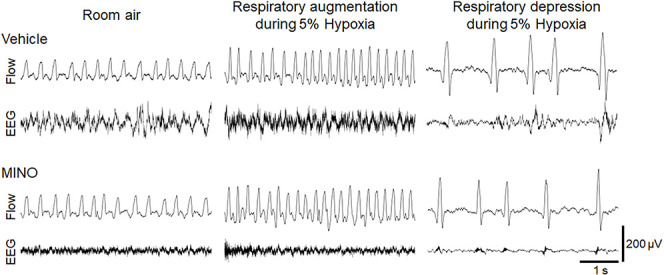
Representative raw recordings of respiratory flow (inspiration upward) and EEG signal in mice without and with minocycline in room air and 5% hypoxia showing the initial ventilatory augmentation and late ventilatory fall-off. Minocycline failed to influence the hypoxic ventilatory response.

**Figure 3:**
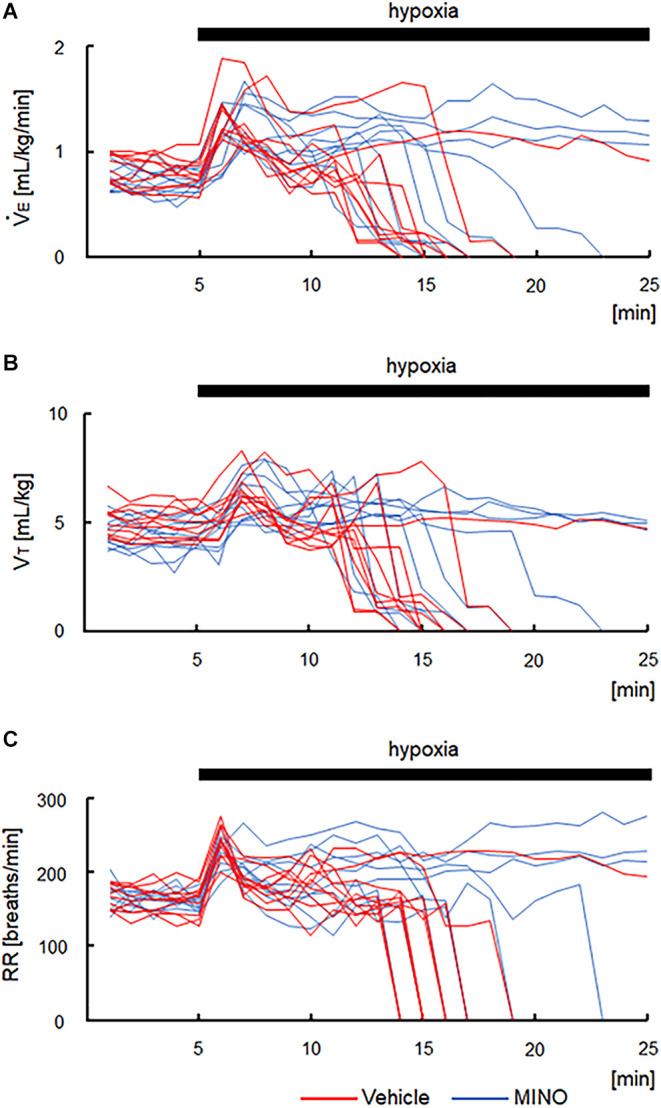
Time courses of respiratory variables in the vehicle- and minocycline pre-treated conditions before and during 5% hypoxia. **(A)** Minute ventilation (⩒_E_). **(B)** Tidal volume (V_T_) in each mouse. **(C)** Respiratory rate (RR). As compared to vehicle-pre-treated mice, minocycline-pre-treated mice tended to resist respiratory depression and respiratory arrest during the late hypoxic phase. MINO, minocycline.

The functional investigation showed that severe hypoxia transiently augmented ventilation followed by the ventilatory fall-off in all mice. Figure 2 shows raw recordings of respiratory flow and EEG. Table 1 presents ⩒_E_, V_T_, and RR in room air and the initial hypoxic augmentation without and with minocycline. Respiration increased in response to hypoxia in both conditions; an increase remained unaffected by minocycline pre-treatment. Statistically, the ANOVA revealed the significant main effect on ⩒_E_ of oxygen condition (*F*(1, 18) = 94.482, *p* < 0.001), no effect of pharmacological condition (*F*(1, 18) = 0.281, *p* = 0.646), and no interaction between pharmacological and oxygen conditions (*F*(1, 18) = 1.243, *p* = 0.280). Changes in V_T_ and RR were in harmony with those in ⩒_E_. Likewise, there were main effects on both V_T_ and RR of oxygen conditions (*F*(1, 18) = 90.816, *p* < 0.001 and *F*(1, 18) = 33.763, *p* < 0.001, respectively), but no significant effects of pharmacological conditions on either variable. The time courses of ⩒_E_, V_T_, and RR in individual mice in the conditions without and with minocycline are shown in Figure 3. There appeared essentially no difference in ventilation between the two conditions in room air and during the early hypoxic phase with respiratory augmentation. However, as compared to the vehicle-pre-treated mice, the minocycline-pre-treated mice tended to resist respiratory depression and respiratory arrest during the late hypoxic phase.

**Table 1:** Respiratory variables in room air and during the respiratory augmenting phase in 5% hypoxia in the two conditions, without and with minocycline pre-treatment.

	⩒_E_ [mL/g/min]		V_T_ [µl/g]		RR [breath/min]	
	Vehicle	Minocycline	Vehicle	Minocycline	Vehicle	Minocycline
Room air	0.82 ± 0.14	0.73 ± 0.12	5.00 ± 0.70	4.47 ± 0.73	163 ± 18	163 ± 14
Hypoxia	1.28 ± 0.26	1.26 ± 0.23	5.30 ± 0.81	6.09 ± 0.97	241 ± 22	207 ± 24

Values are means ± SD. ⩒_E_, minute ventilation. V_T_, tidal volume. RR, respiratory rate. All variables increased during hypoxia compared to room air (*p* < 0.001). Minocycline pre-treatment did not significantly affect the variables.

**Figure 4:**
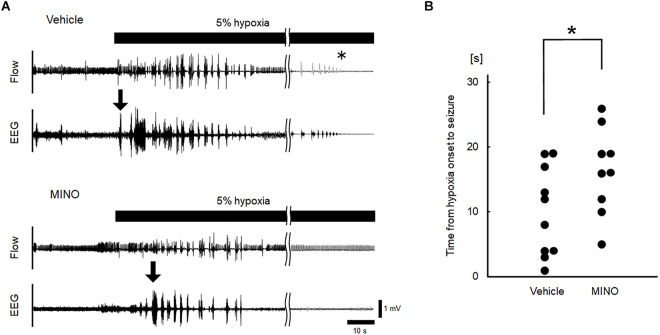
Inhibition of microglial activation delayed the occurrence of seizures. **(A)** Representative raw recordings of respiratory flow (inspiration upward) and EEG signals in hypoxia experiments without and with minocycline. Seizures were accompanied by high amplitude aberrant waves in EEG (the onset of seizures was indicated by downward arrows). The time of respiratory arrest was indicated by an asterisk.** (B)** Time from the onset of hypoxia to seizures in vehicle and minocycline pre-treated mice. Time to seizure was significantly longer in the latter group of mice (Mann-Whitney U test). MINO, minocycline. ^*^p < 0.05.

**Figure 5:**
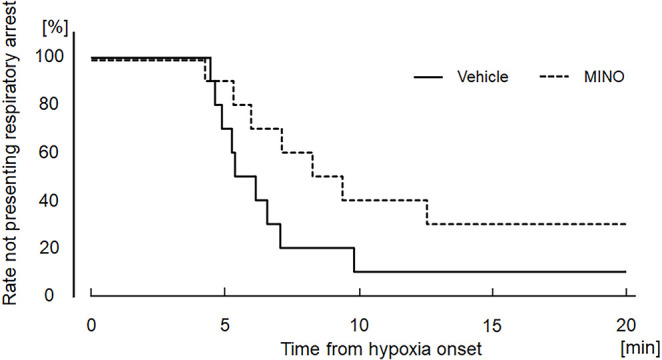
Kaplan−Meier curves showing the time from hypoxia onset to respiratory arrest in the vehicle (saline) and minocycline pre-treated mice; 10 mice each. There were no significant differences between the two groups. MINO, minocycline.

Hypoxia also induced seizures in all but one minocycline-pre-treated mouse. Figure 4A shows representative raw traces of respiratory flow and EEG. At the onset of seizures, characteristic high amplitude aberrant waves in EEG were observed. In the mice without administration of minocycline, theta power, and gamma power were 44.84 ± 31.12 and 0.97 ± 0.75 μV^2^/Hz in room air, 27.19 ± 13.12 and 1.14 ± 1.10 μV^2^/Hz during the time period from hypoxic exposure to seizure appearance, respectively. In the mice pre-treated with minocycline, theta power, and gamma power were 15.59 ± 12.85 and 0.41 ± 0.45 μV^2^/Hz in room air, 8.45 ± 7.37 and 0.43 ± 0.41 μV^2^/Hz during the time period from hypoxic exposure to seizure appearance, respectively. The ANOVA revealed the significant main effect on theta power of pharmacological condition (*F*(1, 17) = 9.472, *p* < 0.01), no effect of oxygen condition (*F*(1, 17) = 1.986, *p* = 0.177), and the interaction between pharmacological and oxygen conditions (*F*(1, 17) = 11.091, *p* < 0.01). The *post-hoc* Welch’s *t*-tests showed a significant reduction in theta power from the vehicle to minocycline administration both in room air (*p* < 0.05) and in hypoxia (*p* < 0.005). The *post-hoc* paired *t*-tests showed a significant reduction in theta power from room air to hypoxia in conditions both without pre-administration of minocycline (*p* < 0.05) and with minocycline (*p* < 0.01). Changes in gamma power were not in harmony with those in theta power. There were no main effects on the gamma power of oxygen conditions (*F*(1, 17) = 0.529, *p* = 0.477), pharmacological conditions (*F*(1, 17) = 3.733, *p* = 0.070), and no interaction between the two (*F*(1, 17) = 0.949, *p* = 0.344). Time to seizure occurrence was significantly longer in the minocycline than in vehicle pre-treated mice (*p* < 0.05; Figure 4B). Following the seizures, mice frequently exhibited respiratory arrests. Figure 5 shows that the minocycline pre-treated mice tended to have fewer respiratory arrests compared to the vehicle pre-treated mice (*p* = 0.12).

## Discussion

Here, we investigated the role of microglia in the appearance of severe hypoxia-induced seizures and post-ictal respiratory arrest. This comparative study used a pharmacologic tool consisting of minocycline to unravel the potential role of microglia in the ventilatory responses to hypoxia and the appearance of seizures. Our immunohistological investigation showed that Iba-1 positive cells converted from the resting ramified to activated de-ramified ovoidal form in hypoxia in the control vehicle pre-treated condition. However, pre-treatment with minocycline inhibited the hypoxia-driven conversion. These results suggest that hypoxia did activate microglia, which was suppressed by minocycline. Severe hypoxia, applied for 20 min, initially increased ventilation, but subsequently caused seizures followed by the post-ictal ventilatory fall-off in all but one mouse. Further, compared to the vehicle group, minocycline pre-treated mice showed a delayed appearance of seizures and tended to resist respiratory arrest.

In the present study, minocycline administration did not affect respiratory variables in room air or the initial hypoxic augmentation. These results are at variance with minocycline-induced reductions in hypoxia-induced increases in ⩒_E_ and V_T_ in rats in the study reported by Silva et al. (2017). However, it should be considered that microglia are positioned upstream of astrocytes. When microglia are activated, they transmit ATP that binds to P2Y1R on astrocytes and activates neighboring astrocytes in a paracrine manner (Pascual et al., 2012). Astrocytes enhance respiratory output by releasing gliotransmitters (Okada et al., 2012; Rajani et al., 2018; Sheikhbahaei et al., 2018). Astrocytes can also act as hypoxia sensors thereby modifying the hypoxic ventilatory response (Tadmouri et al., 2014; Angelova et al., 2015; Fukushi et al., 2016, 2021; Uchiyama et al., 2020). The present results did not support a mediatory role of astrocytes *via* microglia in hypoxic ventilatory augmentation, since minocycline failed to affect respiratory variables. Alternatively, the protocol might not allow sufficient time for the transmission of microglial activation to astrocytes to augment ventilation.

In animal models, activated microglia decrease the seizure threshold by releasing proinflammatory chemokines such as IL-1β or TNF-α, which promotes glutamate release by astrocytes (Volterra and Meldolesi, 2005; Vezzani et al., 2011; Galic et al., 2012; Vezzani et al., 2013; Benson et al., 2015). These effects may be relayed over astrocytes. Pre-treatment with minocycline reduces IL-1β and TNF-α mRNA levels in the rostral ventrolateral medulla after acute hypoxia in rats (Silva et al., 2017). The present findings lend support to scarce animal data showing that brain microglia activated by hypoxia could exert proconvulsive action through the production of IL-1β and TNFα if the suppression of these cytokines by minocycline delayed the onset of seizures.

Microglial and astrocytic activation are well-described features of epilepsy. Dysregulation of astrocytic function contributes to hyperexcitation of neuronal networks leading to seizures (Devinsky et al., 2013; Coulter and Steinhäuser, 2015; Fukushi et al., 2020). Microglial activation occurs before reactive astrogliosis in brain diseases (Sofroniew and Vinters, 2010; Shinozaki et al., 2014). Acute hypoxia activates microglia that enhance astrocytic function through the release of ATP as discussed above. Sano et al. (2021) reported that the sequential activations of microglia and astrocytes are essential for epileptogenesis and susceptibility to seizures in a drug-induced epileptic model. In the present study, hypoxia-induced microglial activation conceivably caused astrocytic activation and induced seizures. The inhibition of microglial activation by minocycline delayed astrocytic excitation and consequently the onset of seizures.

Microglia are activated in human epilepsy, including mesial temporal sclerosis, focal cortical dysplasia, tuberous sclerosis complex, and Rasmussen’s encephalitis (Beach et al., 1995; Boer et al., 2006; Devinsky et al., 2013). Consistent with human studies, microglial activation has been found in animal epileptic brain tissue (Taniwaki et al., 1996; Vezzani et al., 2000; Rosell et al., 2003; Dubé et al., 2010; Zolkowska et al., 2012). In the present study, we found microglial excitation in the piriform cortex of mice under severe hypoxia, which was inhibited by pre-treatment with minocycline (Figure 1). These results suggest that hypoxia-induced microglial excitation in the brain regions critical for epileptogenesis plays a role in the onset of seizures. Minocycline by inhibiting hypoxia-induced microglial excitation would counteract the lowering of the seizure threshold.

In the present study, minocycline administration suppressed theta power in room air and further suppressed it during severe hypoxia. It has been reported that epileptic mice, but not non-epileptic mice, exhibit a decrease in theta power before a seizure (Milikovsky et al., 2017; Mazziotti et al., 2020). Essentially the same finding has been reported in humans (Bettus et al., 2008; Milikovsky et al., 2017). Our results are consistent with these reports. However, the relationship between theta oscillation and seizures remains unclear. Further research should be undertaken to investigate the relationship between EEG power in theta band and seizures. It has also been reported that gamma activity increases before seizures in rodents (Medvedev et al., 2000; Medvedev, 2002; Maheshwari et al., 2016). Our results did not agree with these reports, which may be due in part to the small sample size.

The definition of SUDEP is “sudden, unexpected, witnessed or unwitnessed, non-traumatic and non-drowning death in patients with epilepsy, with or without evidence of a seizure, and excluding documented status epilepticus, where post-mortem examination does not reveal a toxicological or anatomical cause of death” (Nashef, 1997). Dysfunction of the cardiorespiratory autonomic system and sudden death are frequently associated with seizures in chronic epilepsy (Kloster and Engelskjøn, 1999; Langan et al., 2000; So et al., 2000; Opherk et al., 2002; Seyal et al., 2010; Mulkey and Milla, 2022). When patients with epilepsy and respiratory disorders experience hypoxic episodes, microglia-activated neuroinflammatory changes may trigger seizures and cause post-ictal respiratory arrest and SUDEP.

In synopsis, the study suggests that microglia be conducive to seizure activity in severe hypoxia. The finding that minocycline pre-treated mice tended to resist post-ictal respiratory arrest suggests that microglia antagonism could help prevent seizures and SUDEP under severe hypoxia, which would be worthwhile to explore in clinical trials.

## Data availability statement

The raw data supporting the conclusions of this article will be made available by the authors, without undue reservation.

## Ethics statement

The animal study was reviewed and approved by Animal Experiment Ethics Committee of Murayama Medical Center.

## Author contributions

IF conceived and designed the study, performed the animal experiments, analyzed data, and drafted the manuscript. KI performed the immunohistochemical analyses, and drafted the manuscript. KT performed the statistical analysis and drafted the manuscript. YK, MY, and YH participated in the design of the study. MP edited and revised the manuscript. YO conceived and designed the study, analyzed the data, and revised the manuscript. All authors contributed to the article and approved the submitted version.
